# Enantioselective α-heterofunctionalization reactions of catalytically generated C1-Lewis base enolates

**DOI:** 10.1016/j.tchem.2024.100063

**Published:** 2024-03

**Authors:** Magdalena Piringer, Lotte Stockhammer, Lukas Vogl, David Weinzierl, Paul Zebrowski, Mario Waser

**Affiliations:** Institute of Organic Chemistry, Johannes Kepler University Linz, Altenbergerstr. 69, 4040 Linz, Austria

**Keywords:** Organocatalysis, Covalent catalysis, Lewis bases, Heterofunctionalizations, Carboxylic acid derivatives

## Abstract

Chiral Lewis base (LB) organocatalysis has emerged as a powerful covalent catalysis concept which allows for highly selective asymmetric C–C and C-heteroatom bond formations. Considering significant recent progress in the development of strategies to access α-heterofunctionalized carboxylic acid derivatives under chiral LB catalysis, we wish to summarize the most significant concepts and advances in this field within this mini review now.

## 1 Introduction

**Scheme 1:**
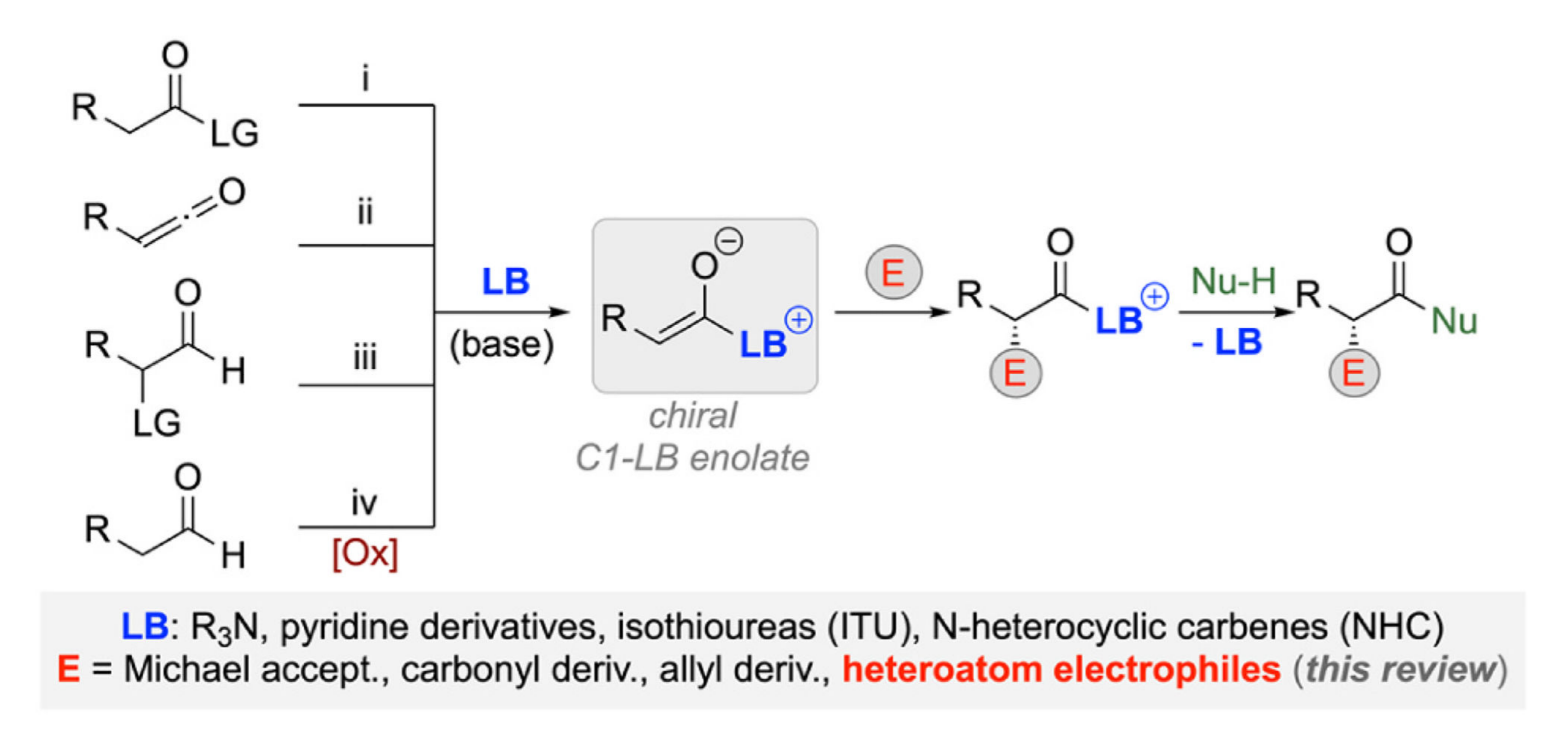
In situ generation and subsequent utilization of chiral C1-LB enolates.

Chiral Lewis base (LB) organocatalysis has emerged as a powerful concept within the realm of asymmetric catalysis [1–7]. Upon in situ addition of a LB to electron deficient Lewis acidic groups of starting materials and reagents (most commonly via n(LB) -> π*(reagent) interactions) activated chiral intermediates are obtained, which then allow for unique transformations with often excellent levels of stereo-control. Different classes of Lewis bases have been successfully established for the activation and control of various starting materials and reaction partners [1–7], thus resulting in a broad and generally applicable catalysis concept that has been carefully reviewed in numerous excellent overview articles over the last two decades [2–4,7]. Amongst the different classes of compounds that can be successfully engaged in asymmetric LB-catalyzed transformations, carbonyl compounds and carboxylic acid derivatives turned out to be especially rewarding, allowing for the use of easily accessible starting materials for highly appealing transformations. The utilization of aldehydes and ketones in combination with chiral LBs most usually proceeds via iminium and enamine intermediates (when using 1° or 2° amines) [8] or Breslow type intermediates (using N-heterocyclic carbenes (NHCs) [9]. On the other hand, the use of activated carboxylic acid derivatives (i), ketenes (ii), carbaldehydes with a leaving group in the α-position (iii), or carbaldehydes under oxidative conditions (iv) in combination with LBs such as 3° amines, pyridine derivatives, isothioureas (ITU), or NHCs proceeds via the formation of so-called C1-LB enolates (Scheme 1) [2–4]. These species benefit from their high nucleophilicity and a well-defined enolate geometry, thus combining high reactivity with usually high levels of stereocontrol for α-functionalization reactions. Following the stereo-defining addition to an electrophilic species, the covalently bound LB can be released upon addition of either an (external) nucleophile in a bimolecular reaction or via an intramolecular cyclization path (in case a dipolar reaction partner has been utilized). In general, the field of C1-LB enolate chemistry i.e. when using 3° amine catalysts or pyridine-based catalysts (giving C1 ammonium enolates) has a long-standing history dating back to Pracejus’ seminal report describing the Cinchona alkaloid-catalyzed asymmetric methanolysis of ketenes proceeding via an in situ formed C1 enolate [10]. While initially these C1 enolates have primarily been engaged in C–C bond forming reactions or ketene solvolysis approaches [2], it was also soon realized that such species serve well for asymmetric α-heterofunctionalization reactions [11,12]. Considering significant progress in the development of asymmetric approaches to access valuable α-heterofunctionalized carboxylic acid derivatives under chiral LB catalysis, we herein wish to summarize the most significant concepts and advances in this field. Our main focus hereby lies on strategies where the catalytically generated chiral C1-LB enolate is engaged in an asymmetric Cα-heteroatom bond formation, while approaches relying on e.g. asymmetric protonations of C1 ammonium enolates [13], resolutions of racemic α-heterofunctionalized compounds [14], asymmetric C–C-bond forming reactions of prochiral α-heterofunctionalized derivatives [15], or asymmetric [2, 3]-rearrangements [16] are not within the scope of this mini-review.

## 2 α-Halogenations

Asymmetric α-halogenations [17] have clearly been the most thoroughly investigated and furthest developed α-heterofunctionalization reactions of in situ generated chiral C1-LB enolates. The value of the hereby obtained α-halogenated carboxylic acid derivatives can on the one hand be attributed to potentially interesting biological properties associated either with these carboxylic acid derivatives themselves, or with more complex halogenated structures accessed therefrom (this comes especially true for fluorinated derivatives) [17]. On the other hand, chiral α-chlorinated or α-brominated carboxylic acid derivatives serve well for further stereospecific S_N_2 displacements with various nucleophiles, thus representing valuable chiral building blocks for the synthesis of compounds where the direct asymmetric installation of the target group is problematic.

**Scheme 2:**
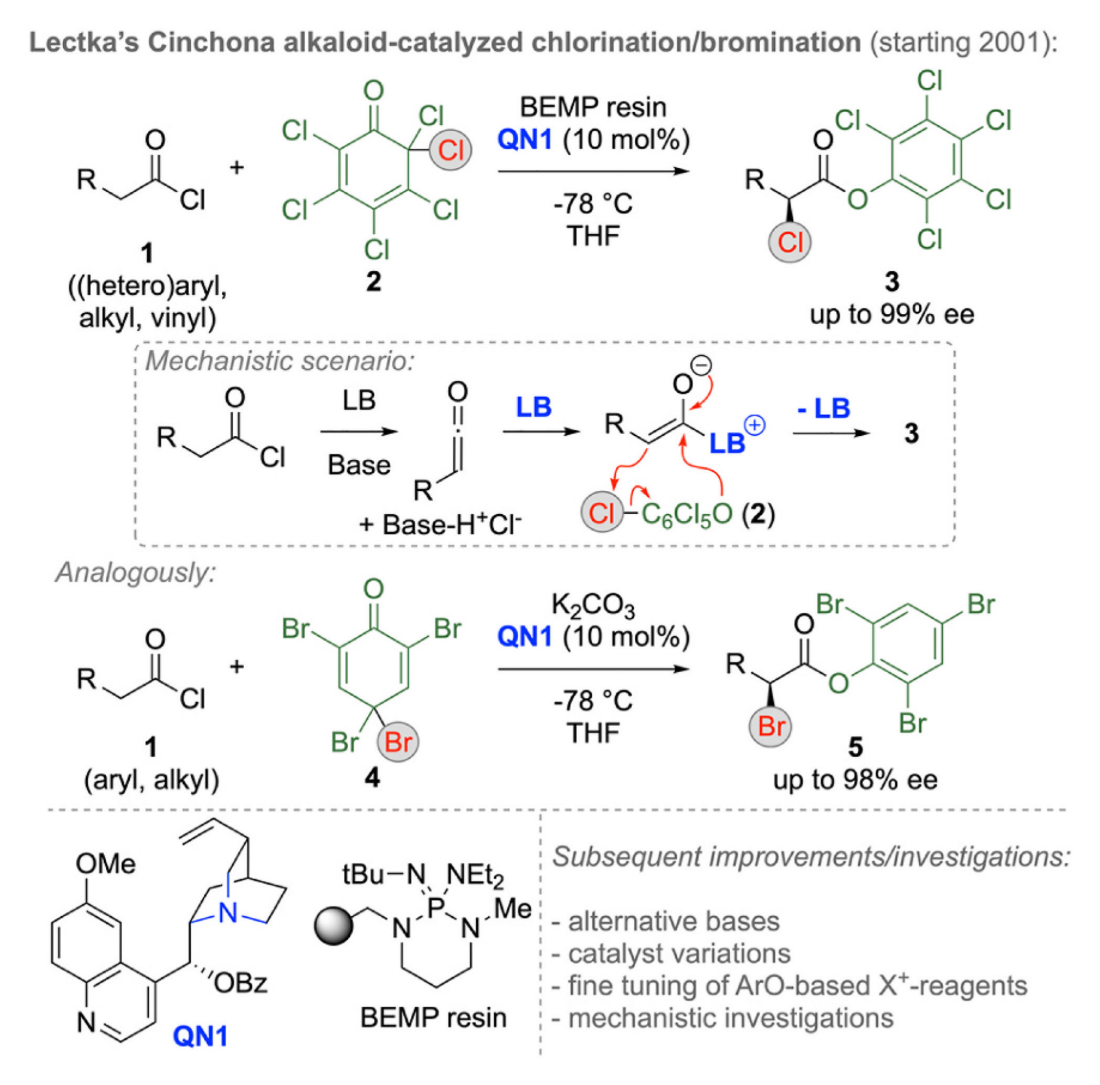
Lectka’s pioneering Cinchona alkaloid-catalyzed α-halogenation reports of in situ formed ketenes.

Pioneering early contributions on asymmetric α-halogenations of C1-LB enolates were reported by Lectka’s group around two decades ago (Scheme 2) [12]. Upon utilizing acyl halides **1** as pronucleophiles and cyclohexadienone **2** as an electrophilic Cl-transfer reagent in the presence of Cinchona alkaloids (e.g. **QN1**) as chiral Lewis base catalysts and an external Brønsted base they managed to access the α-chlorinated aryl esters **3** with high yields and excellent levels of enantioselectivities. Mechanistically, this transformation proceeds via in situ ketene formation which, upon LB-addition, gives the crucial chiral C1 enolate which then adds to reagent **2** first, followed by “back-biting” and replacement of the LB by the in situ generated phenolate species. This conceptually impressive approach where the carefully chosen electrophilic chlorine transfer reagent is not only relevant for the α-Cl-installation but also forms a nucleophilic species (a phenolate) in situ represents a remarkable development for the whole field as it allows for efficient catalyst turnover. Furthermore, the hereby accessed activated aryl esters **3** were successfully utilized for further manipulations like direct amide-bond formations, which underscores the synthetic versatility of these products. Interestingly, the nature of the external base was found to be crucial in order to allow for efficient ketene-formation and to suppress by-product formation and non-productive pathways like e.g. ketene hydrolysis. In their initial studies, Lectka’s team used a solid-supported phosphazene base (BEMP resin). Later studies however showed that other bases (e.g. NaH/crown ether combinations) and modified Cl-transfer reagents can be successfully utilized as well [18]. These pioneering developments, which were also accompanied by very helpful mechanistic studies, set the stage for a variety of further developments in the field of C1-LB enolate chemistry and, in addition to these α-chlorinations, analogous α-brominations were successfully developed as well. Hereby electrophilic Br-transfer reagents like compound **4** were found to be well-suited, being again capable of forming the crucial phenolate species that allows for catalyst turnover in situ [12,18].

**Scheme 3:**
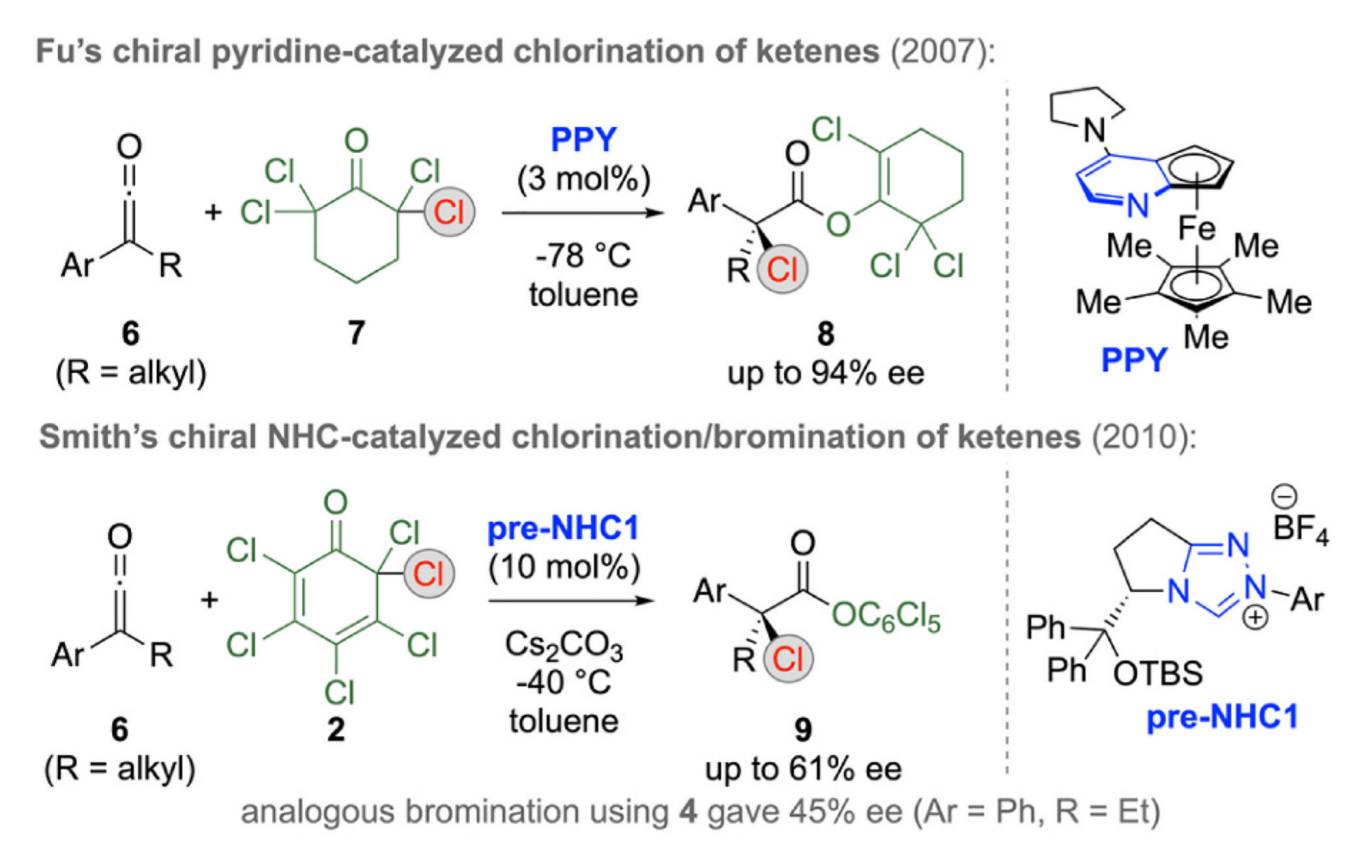
Lewis base-catalyzed α-halogenations of α,α-disubstituted ketenes **6**.

A few years after Lectka’s seminal reports utilizing halides **1** as α-substituted ketene precursors (Scheme 2), Fu’s group reported the direct utilization of α,α-disubstituted ketenes **6** for α-chlorinations with ketone **7** in the presence of their planar chiral pyridine-based LB catalyst **PPY** (Scheme 3, upper reaction) [19a]. Remarkably, this approach gives access to tetrasubstituted chlorinated stereocenters with high levels of enantioselectivities upon using low amounts of catalyst **PPY** only and further manipulations of the ester functionality can easily be carried out as well. Shortly after, Smith’s group utilized in situ generated NHCs (e.g. starting from **pre-NHC1**) as LB catalysts for analogous reactions, in this case however relying on Cl-reagent **2** (Scheme 3) [19b] and some years later chloral was also successfully used as an alternative Cl-transfer re-agent for such reactions [19c].

**Scheme 4:**
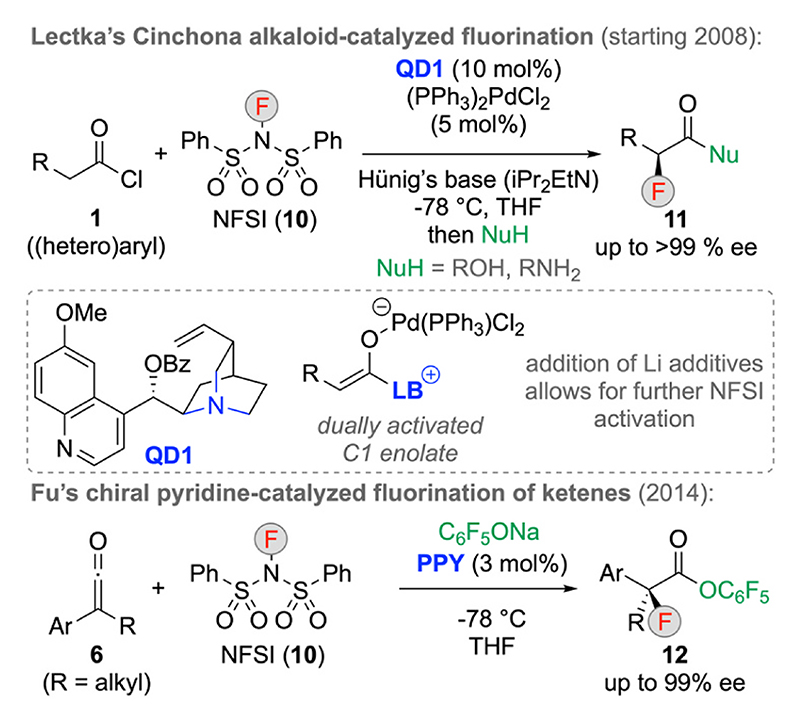
Asymmetric α-fluorination approaches of in situ (Lectka) or preformed (Fu) ketenes.

A few years after introducing their α-chlorination and α-bromination strategies via in situ formed ketenes (Scheme 2), Lectka’s group also reported analogous α-fluorination reactions (Scheme 4, upper part) [20]. However, some important changes and optimizations as compared to their Cl/Br-transfer protocols were necessary. First of all, the concept of using electrophilic halogen-transfer reagents which form the required nucleophilic species that allow for catalyst turnover in situ is not feasible hereby, as established F^+^-reagents (like NFSI) usually only form weakly nucleophilic species after the F-transfer. Thus, reaction conditions employing external nucleophiles (like alcohols or amines) had to be established. In addition, it was found that the addition of Pd-complexes like (PPh_3_)_2_PdCl_2_ has a very positive effect on the overall performance resulting in significantly enhanced yields. This can be explained by formation of a highly reactive dually activated C1 enolate. In detailed and mechanistically driven follow-up studies it was even shown that the addition of Li additives leads to further activation by increasing the reactivity of the NFSI, thus resulting in an impressive triple-activation strategy [20].

Using preformed ketenes **6** again, Fu’s group was able to successfully extend their **PPY**-catalyzed chlorination strategy (Scheme 3) to α-fluorinations next (Scheme 4, lower part) [21]. Hereby, catalyst turnover was successfully achieved by using C_6_F_5_ONa as an external nucleophile, which gives access to the α-fluoro esters **12** with excellent enantiose-lectivities and further ester manipulations were successfully reported again.

**Scheme 5:**
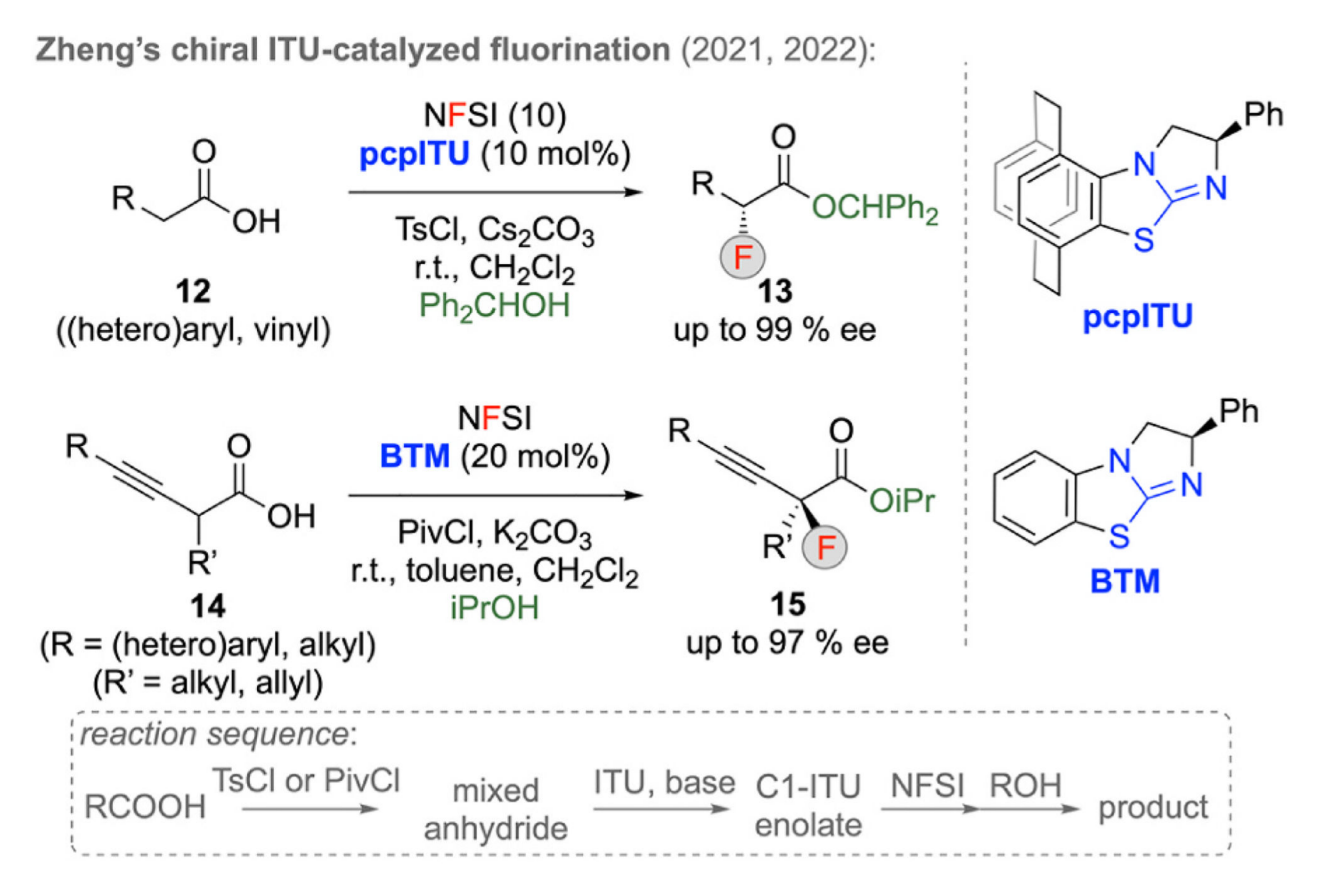
Isothiourea-catalyzed α-fluorination of in situ activated carboxylic acid derivatives.

Besides the already mentioned Cinchona alkaloids, pyridine-based-catalysts, and NHCs, also isothioureas (ITUs) have, over the course of the last years, been very successfully used for asymmetric C1-LB enolate chemistry [3]. These catalysts are most commonly employed in combination with activated carboxylic acid derivatives (either preformed or in situ formed) which, in the presence of an external base, gives the geometrically well-defined chiral C1-ITU enolate in situ [3]. Surprisingly however, the potential of these catalysts to facilitate asymmetric α-halogenations has only recently been established [22,23]. In 2021, Zheng’s group introduced the para-cyclophane-based ITU **pcpITU** which was successfully used for the α-fluorination of free carboxylic acids **12** (Scheme 5) [22a]. Hereby, acid **12** is in situ activated by using TsCl to form the mixed anhydride. This species then reacts with the LB and upon deprotonation gives the chiral C1 enolate which adds to NFSI. Catalyst turnover was hereby achieved by addition of alcohols, thus giving access to the α-fluorinated esters **13** containing a tri-substituted stereogenic center. Furthermore, this in situ acid-activation – fluorination strategy could successfully be expanded to alkyne-based acids **14**, which allow for the synthesis of α-F-esters **15** with a tetrasubstituted stereogenic center [22b]. Hereby, the authors relied on **BTM** as a readily available ITU derivative instead and isopropanol was found to be the alcohol of choice allowing for good yields and catalyst turnover.

**Scheme 6:**
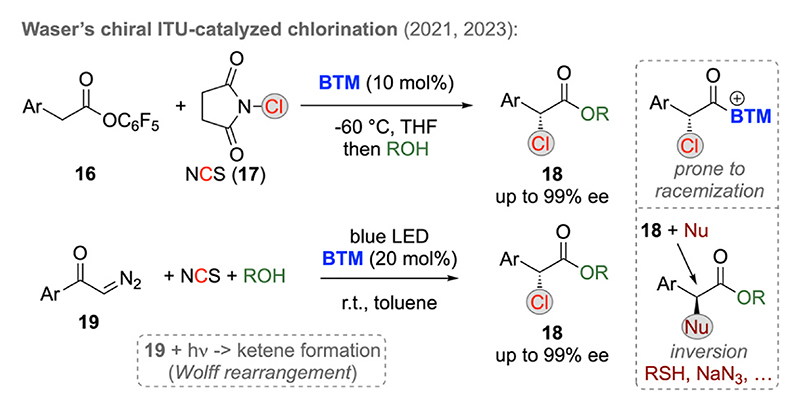
Isothiourea-catalyzed α-chlorination of activated esters and in situ generated ketenes.

More or less in parallel to Zheng’s contributions, our group focused on ITU-catalyzed α-chlorination reactions using NCS as an easily available electrophilic Cl-transfer reagent (Scheme 6) [23]. Instead of starting from free carboxylic acids **12** or **14** we employed electron deficient aryl esters **16** as starting materials first [23a]. We hereby found it crucial to carry out the **BTM**-catalyzed reaction of **16** with NCS and the sub-sequent alcohol-quench under cryogenic conditions, to avoid racemization of the intermediate catalyst-bound α-chlorinated species. Employing this strategy, a variety of α-Cl esters **18** could be obtained straightforwardly. Investigating the use of alternative starting materials next, we recently developed a photochemical protocol starting from α-diazoketones **19** [23b]. Under blue LED irradiation these compounds undergo a Wolff rearrangement forming the corresponding ketene. This ketene is then trapped by the ITU followed by subsequent α-chlorination and ROH addition to release the catalyst again (in analogy to the other ketene approaches discussed above already). Hereby we also demonstrated the suitability of products **18** for stereospecific S_N_2 reactions with heteroatom nucleophiles, which gives access to various α-hetero-functionalized esters with high levels of stereocontrol.

**Scheme 7:**
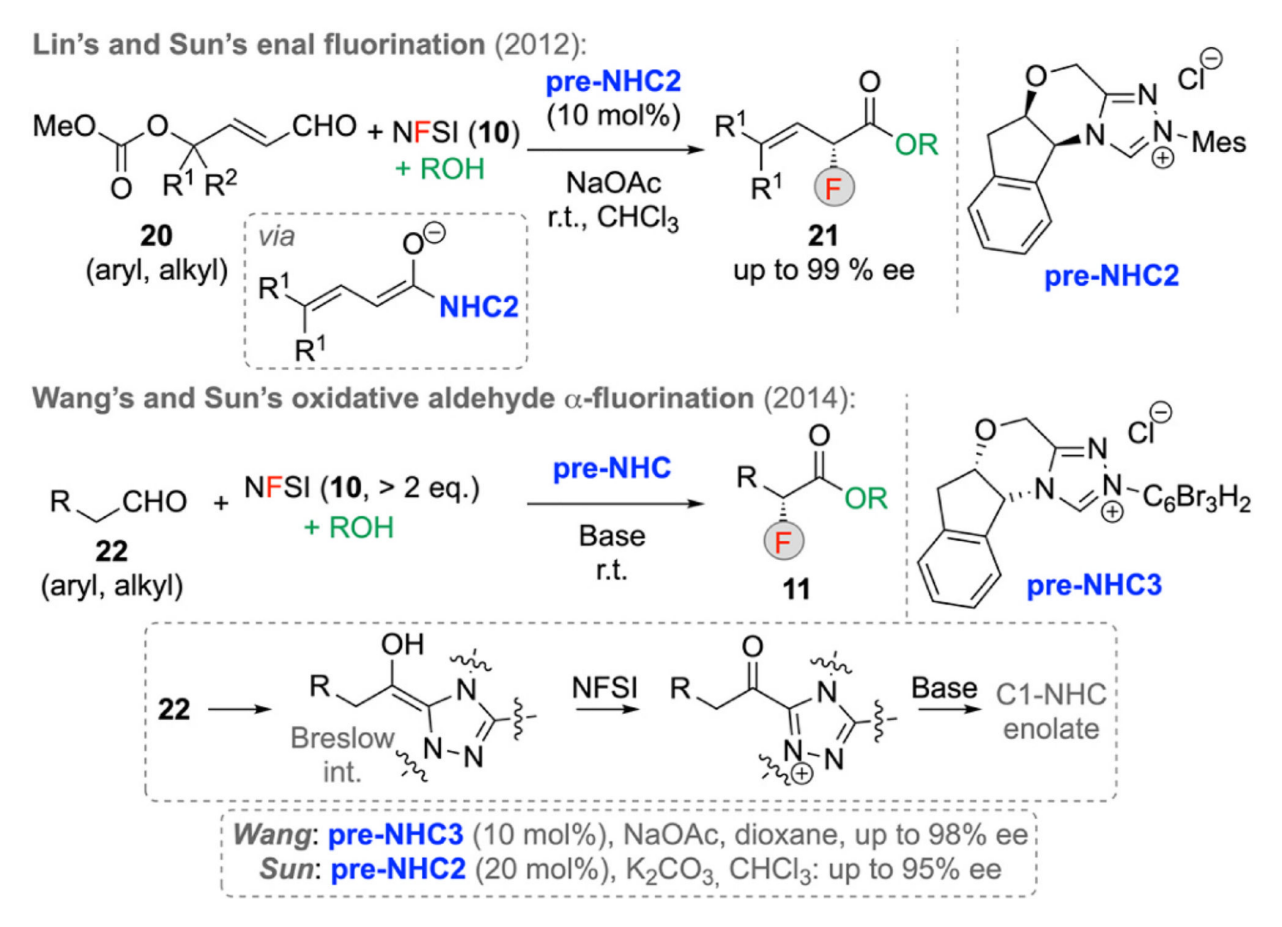
Chiral NHC-catalyzed fluorinations starting from carbaldehydes.

As stated in the introductory part, C1-LB enolates can not only be accessed from ketenes and carboxylic acid derivatives, but also from carbaldehydes. In this case, it is possible to start from carbaldehydes under oxidative conditions, from derivatives that contain a suited leaving group in the α-position or, as impressively shown by Lin and Sun around 10 years ago, by using enals **20** with a leaving group in the γ-position [24a]. Utilizing these starting materials in the presence of the chiral NHC generated from **pre-NHC2** allows for the formation of the α-fluorinated β,γ-unsaturated esters **21** with excellent enantioselectivities. Mechanistically, aldehydes **20** and the NHC catalyst first form a conjugated C1–NHC enolate species which then reacts with NFSI in an asymmetric fashion followed by methanolysis to release the catalyst again (Scheme 7, upper transformation). Alternatively, simple carbaldehydes **22** can also be successfully utilized for the syntheses of α-F-esters **11** when using chiral NHCs in the presence of an excess of NFSI [24b,c]. Hereby the NFSI plays a dual role, as it not only serves as the electrophilic F-transfer reagent, but it also oxidizes the initially formed Breslow intermediate giving the required C1-LB enolate, which then reacts further in the established manner (Scheme 7, lower part).

## 3 Chalcogenations

Asymmetric α-chalcogenations, i.e. α-oxygenations of prochiral enolate species are important transformations to access valuable chiral building blocks. Surprisingly, despite a lot of recent advancements in the introduction of different catalysis concepts to access enantioenriched α-heterofunctionalized carbonyl compounds [11,25], asymmetric α-chalcogenations of C1-LB enolates have so far been less thoroughly developed. One challenge of such approaches so far is the identification of suited reactive electrophilic chalcogen-transfer reagents that allow for asymmetric induction and either trigger the subsequent catalyst turnover themselves or which do not interfere with other nucleophiles that are required for catalyst turnover (please compare with the different successfully developed catalyst turnover strategies discussed above).

**Scheme 8:**
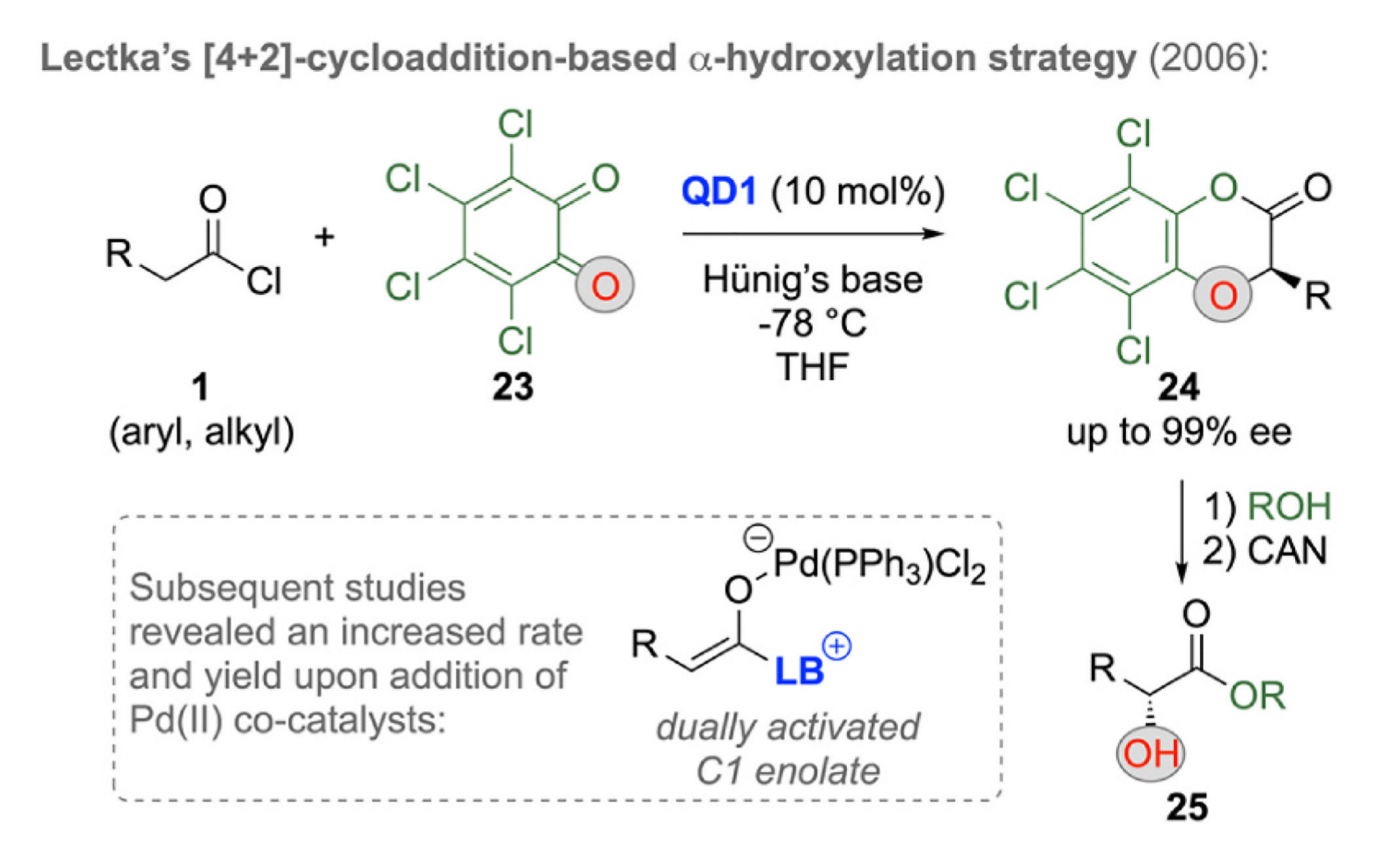
Chiral Cinchona alkaloid-catalyzed hetero-[4 + 2]-cycloadditions delivering α-oxygenated carboxylic acid derivatives.

Pioneering contributions were again reported by Lectka’s group, who first described the Cinchona alkaloid-based α-oxygenation of acyl halides **1** with o-benzoquinone **23** in 2006 (Scheme 8) [26a]. This approach proceeds via ketene-formation/C1-LB enolate formation first and subsequent hetero-[4 + 2]-cycloaddition with **23**, providing the bicyclic lactones **24** with excellent enantioselectivities. Compounds **24** can then be successfully converted into α-hydroxy esters **25** via a two-step process involving lactone hydrolysis (addition of alcohols) and subsequent oxidative cleavage off the α-phenol ether functionality by using ceric ammonium nitrate (CAN). Remarkably, in follow-up studies [26b] the authors realized for the first time that the addition of Pd(II) compounds allows for significantly increased reaction rates and yields, which was attributed to the formation of the doubly activated C1-LB enolates, a concept that was later on then very successfully utilized for the above discussed LB-catalyzed α-fluorination reactions as well (Scheme 4) [20].

**Scheme 9:**
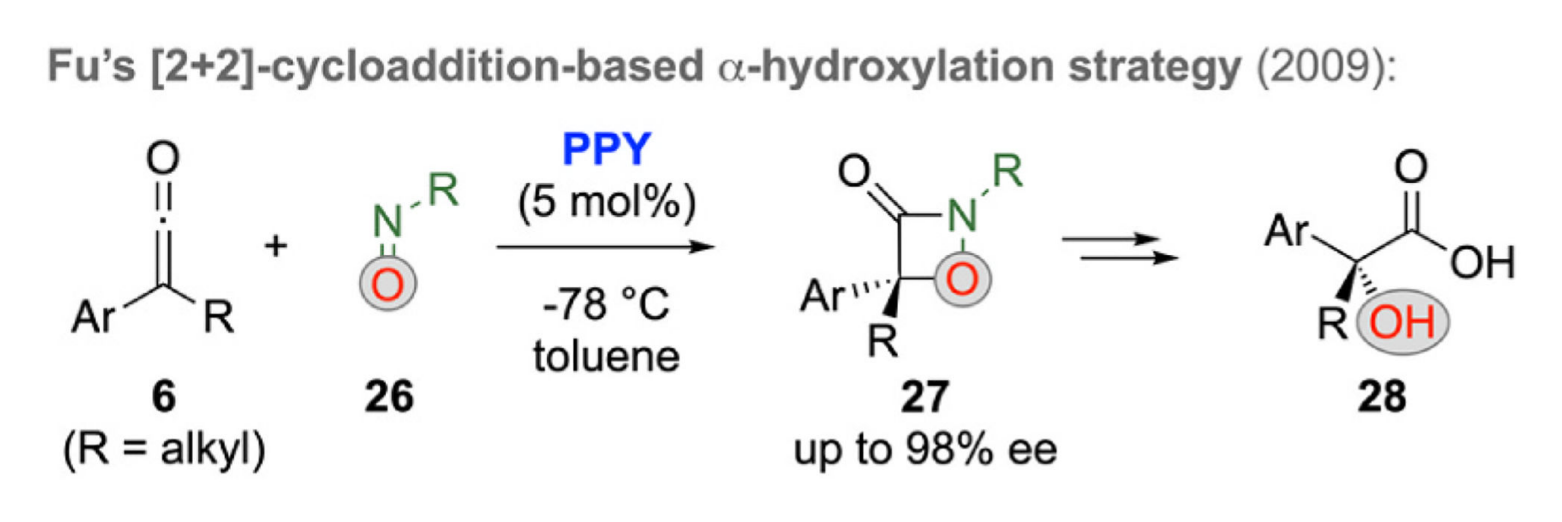
Chiral pyridine-catalyzed synthesis of α-oxygenated carboxylic acid derivatives.

In 2009, Fu’s group expanded their chiral pyridine-based ketene-activation strategy (compare with Schemes 3 and 4) to the hetero-[2 + 2]-cycloaddition of ketenes **6** with nitroso compounds **26** (Scheme 9) [27]. This strategy allows for the highly enantioselective synthesis of 1, 2-oxazetidin-3-ones **27** first, which can then be successfully utilized to access α–OH–carboxylic acid derivatives **28** straightforwardly.

**Scheme 10:**
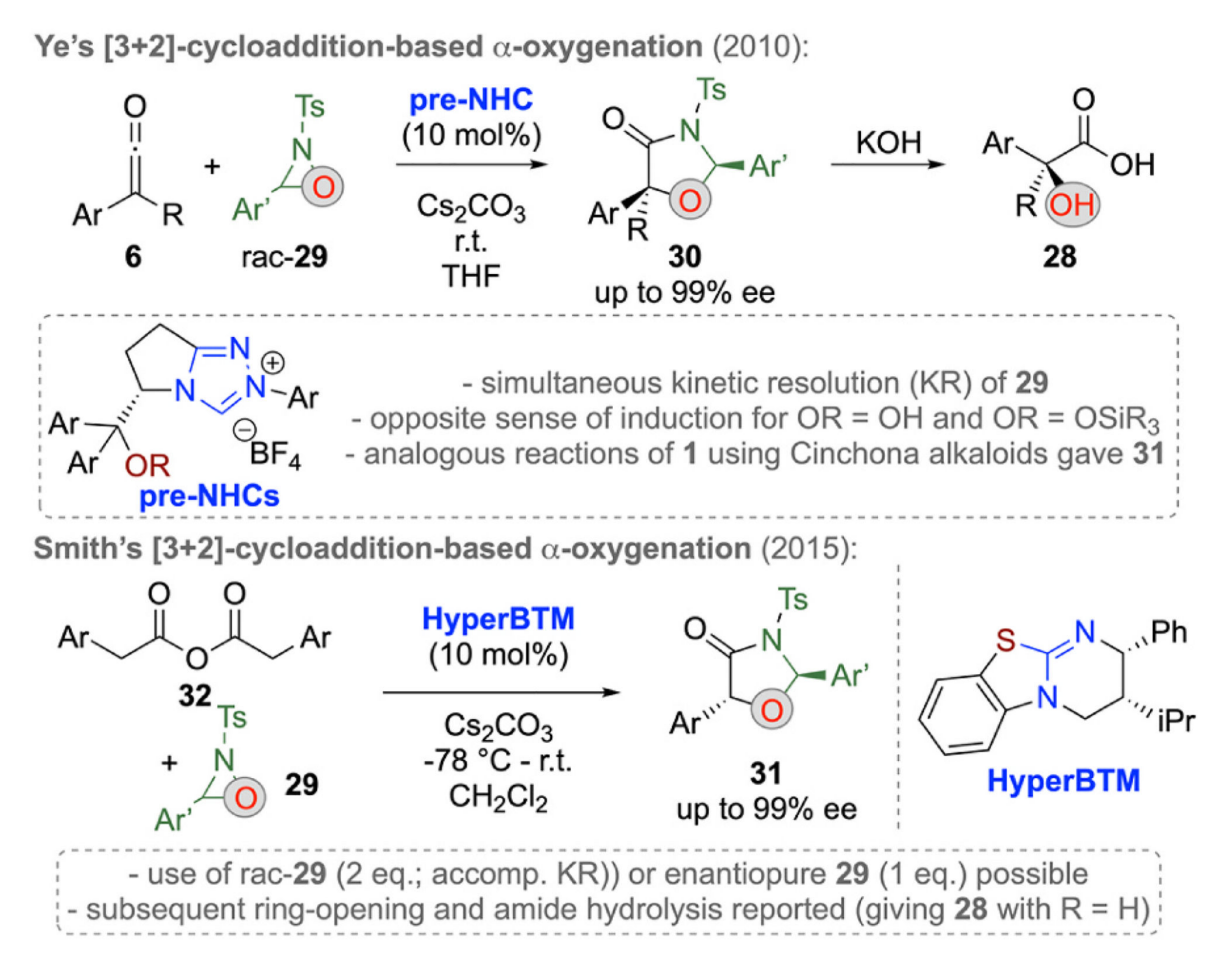
Chiral C1-LB enolate [3 + 2]-cycloadditions with oxaziridines **29**.

N-sulfonyl oxaziridines like compounds **29** are well-established electrophilic O-transfer reagents which have been successfully used for α-hydroxylation reactions of various pronucleophiles [28]. In 2010, Ye’s group first succeeded in utilizing these versatile reagents for asymmetric formal [3 + 2]-cycloadditions with ketenes **6** under chiral NHC catalysis (Scheme 10, upper reaction) [29]. Remarkably, under carefully optimized conditions they managed to utilize racemic oxaziridines **29** for the synthesis of the cyclic α-oxygenated carboxylic acid derivatives **30** with high levels of diastereo- and enantioselectivities. Simultaneously, an efficient kinetic resolution of the mismatching oxaziridine enantiomer was observed as well. Remarkably, the sense of asymmetric induction was depending on the OR-group of the catalyst and either of the two enantiomers of products **30** could easily be accessed by switching from free OH-containing NHCs to OSiR_3_-protected derivatives. Furthermore, cyclic compounds **30** could easily be hydrolyzed to the free α-hydroxy carboxylic acids **28** containing a tetra-substituted stereogenic center. Noteworthy, in this contribution Ye and co-workers also demonstrated that analogous tri-substituted targets **31** (with R═H) can be accessed as well by starting from acyl halides **1** in the presence of chiral Cinchona alkaloid catalysts.

A few years after, Smith’s group reported the use of symmetric anhydrides **32** for analogous [3 + 2]-cycloadditions (Scheme 10, lower reaction) [30]. Hereby chiral ITUs, i.e. **HyperBTM**, were successfully used as chiral Lewis base catalysts. Interestingly, these transformations can either be carried out by using 2 equiv. of racemic oxaziridines **29** proceeding with a simultaneous oxaziridine resolution, or by using a stoichiometric amount of enantiopure **29**. Very recently, David’s and Schulz’s groups also reported the successful use of immobilized ITUs for such transformations, thus allowing for easy recovery of the chiral catalyst [31].

**Scheme 11:**
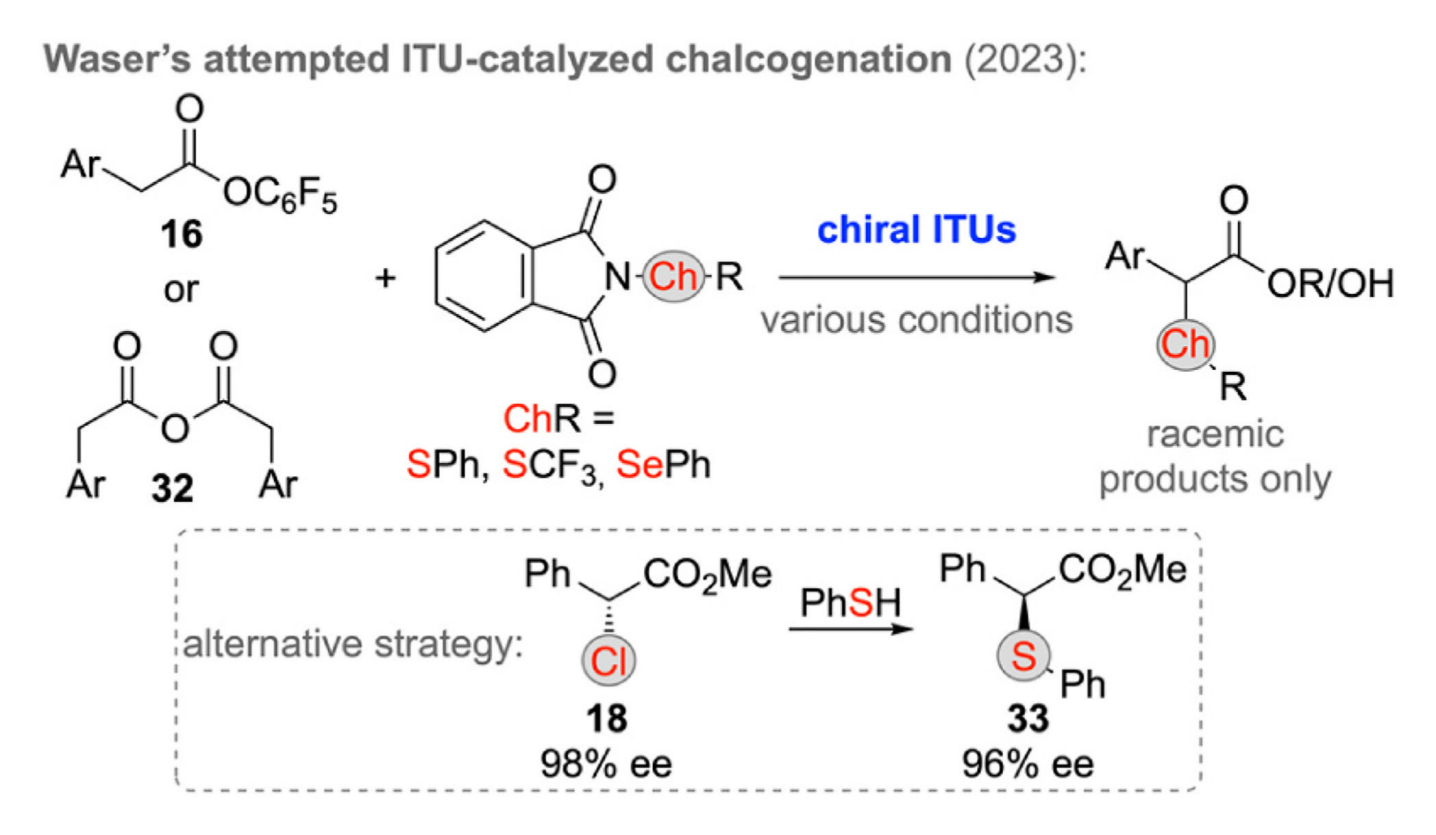
Attempted asymmetric α-sulfanylation and α-selenation reactions.

Surprisingly to us, besides these α-oxygenation reports no further successful enantioselective α-chalcogen approaches of C1-LB enolates have been described so far, at least to the best of our knowledge. Thus, our group recently investigated the potential of LB ammonium enolate chemistry to facilitate asymmetric α-sulfanylation and α-selenation reactions as outlined in Scheme 11. We focused on chiral ITU catalysis in combination with either activated aryl esters **16** or the symmetric anhydrides **32** to carry out α-chalcogenations with different electrophilic S (e)R-transfer reagents. Unfortunately however, despite of an extensive screening of different catalysts and conditions (solvents, nucleophilic quenching reagents, …) we only obtained racemic α-S(e)R-containing esters or carboxylic acids, thus clearly illustrating the limits of the existing methods. A possible alternative strategy to access such targets in an enantioenriched manner is presented in Scheme 6 as we recently found that enantioenriched α-Cl-esters **18** can undergo stereospecific nucleophilic displacement reactions with e.g. S-nucleophiles [23b], which allows for the asymmetric synthesis of targets (like compound **33**) that are not yet accessible with direct electrophilic C1-LB enolate α-heterofunctionalization reactions.

## 4 Aminations

**Scheme 12:**
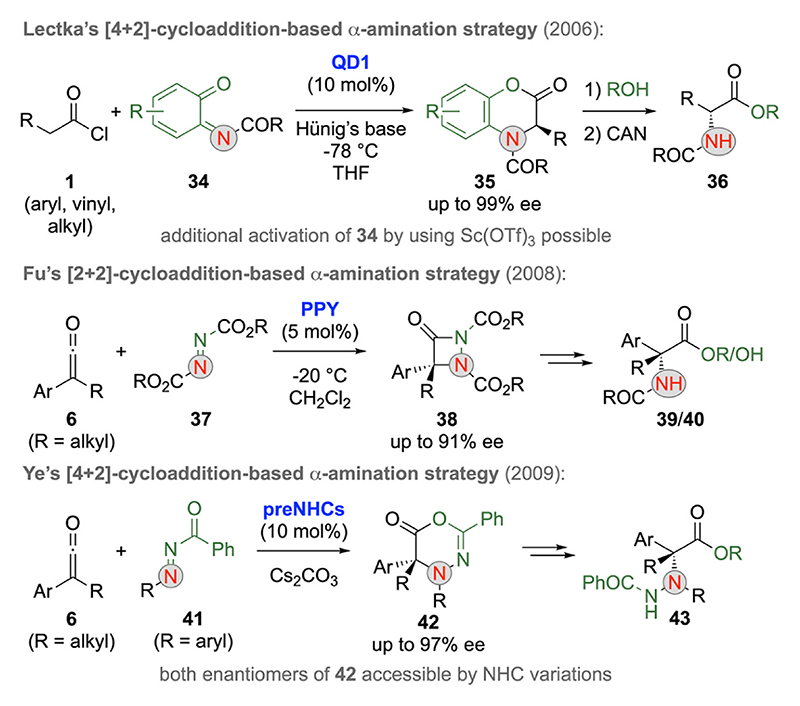
Chiral LB-catalyzed [4 + 2]-cycloaddition (Lectka, Ye) and [2 + 2]-cycloaddition (Fu) strategies using ketenes to access α-amino carboxylic acid derivatives.

The asymmetric catalytic synthesis of α-aminated carbonyl derivatives via electrophilic α-amination reactions of prochiral enolate species is an important task [25]. As for the above-mentioned halogenations and chalcogenations, the successful development of C1-LB enolate-based α-aminations very much requests carefully chosen electrophilic N-transfer reagents and suited strategies for catalyst turnover. Seminal contributions in this field again stem from Lectka’s group [32], who successfully utilized o-benzoquinone imides **34** (and analogous diimides [32a]) for asymmetric [4 + 2]-cycloadditions with acyl halide **1** in 2006 already [32b]. This approach, which again proceeds via in situ ketene formation and subsequent C1-Cinchona enolate formation (compare with Scheme 2), provides a straightforward entry to the chiral 1,4-benzoxazinones **35**, which can then undergo ring opening and oxidative N-aryl cleavage towards the α-amino esters **36** (Scheme 12, upper example) [32b]. Subsequent studies also showed that the addition of Lewis acids, like Sc(OTf)_3_, allows for additional activation of electrophile **34**, thus increasing yield and conversion rate [32c].

Shortly after, Fu’s group reported the [2 + 2]-cycloaddition of ketenes **6** with diazodicarboxylates **37** in the presence of chiral pyridine catalysts [33]. Hereby, the azalactames **38** are formed first (Scheme 12, 2nd reaction), which can then be engaged in several follow-up transformations, like the ring opening hydrolysis to α-amino acids or analogous esters **39** and **40**. The activation of ketenes **6** can also be achieved by using NHCs, as demonstrated by Ye’s group who showed that the reaction of **6** and acceptor **41** allows for the enantioselective synthesis of the [4 + 2]-cycloaddition products **42** (Scheme 12, lower reaction) [34]. Noteworthy, the sense of induction of this cycloaddition can be steered by using NHC-precursors which possess the same absolute configuration but different substitution patterns [34].

**Scheme 13:**
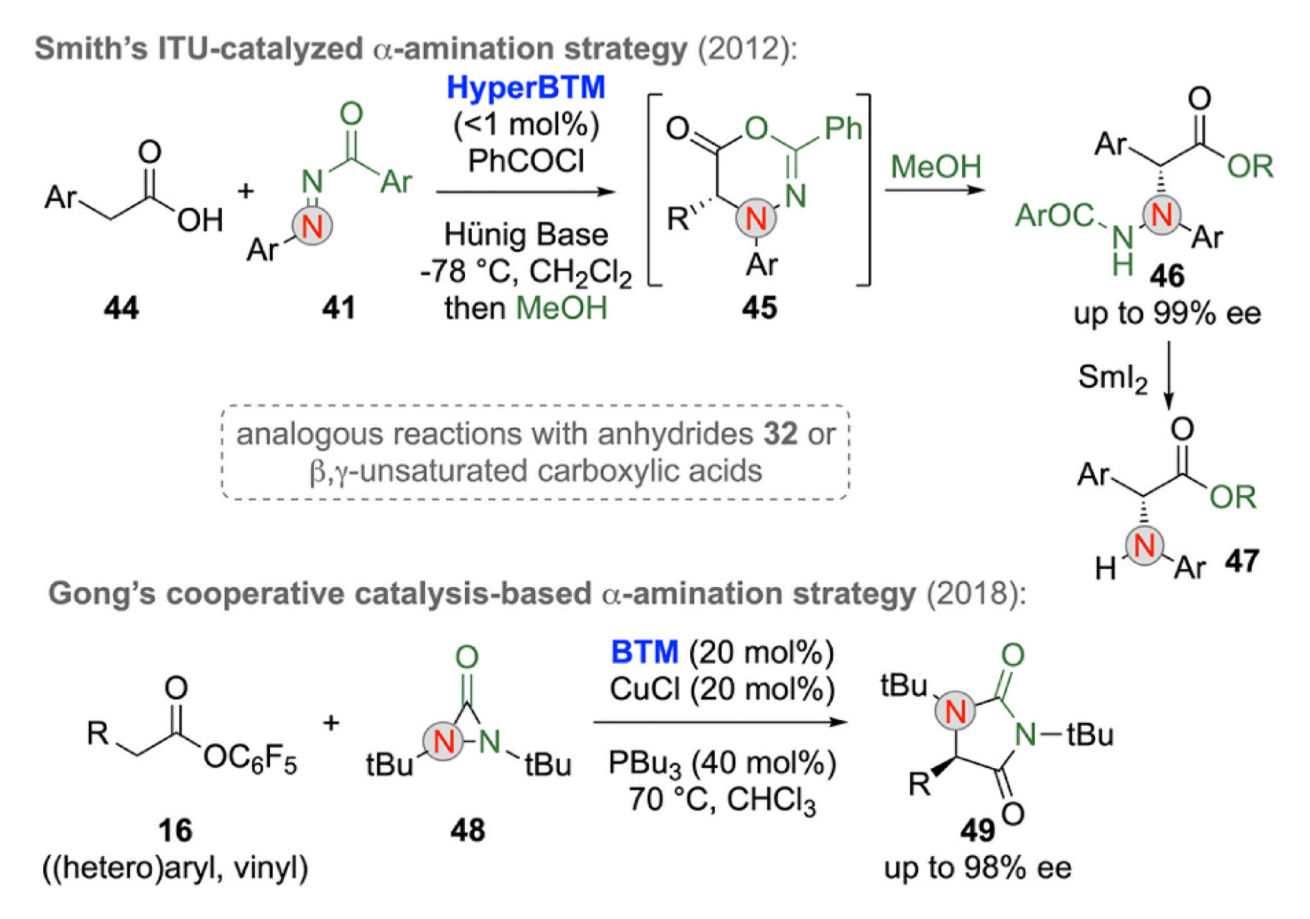
ITU-catalyzed α-amination strategies.

The use of ITUs for asymmetric α-amination reactions was first reported by Smith’s group in 2012 (Scheme 13, upper reaction) [35]. Starting from arylacetic acids **44**, which can be in situ activated using benzoylchloride, the reaction with compounds **41** in the presence of chiral ITUs proceeds via a [4 + 2]-cycloaddition delivering the primary reaction product **45** which can directly be hydrolyzed to ester **46** upon addition of MeOH. Noteworthy, the subsequent SmI_2_-mediated reductive N–N-cleavage of compounds **46** to α-amino esters **47** was successfully demonstrated as well. In follow-up studies, the Smith group also showed that analogous reactions can be carried out with high selectivities by starting from either symmetric anhydrides **32** or β,γ-unsaturated carboxylic acids as well [35].

Recently, Gong and co-workers introduced a highly enantioselective [3 + 2]-cycloaddition of activated esters **16** and diaziridinone **48** (Scheme 13, lower reaction) [36]. Hereby an elegant cooperative catalysis approach using BTM to activate compound **16** and CuCl to activate **48** was developed, thus providing a very efficient and highly enantioselective catalytic protocol to access hydantoins **49** in a straightforward manner.

**Scheme 14:**
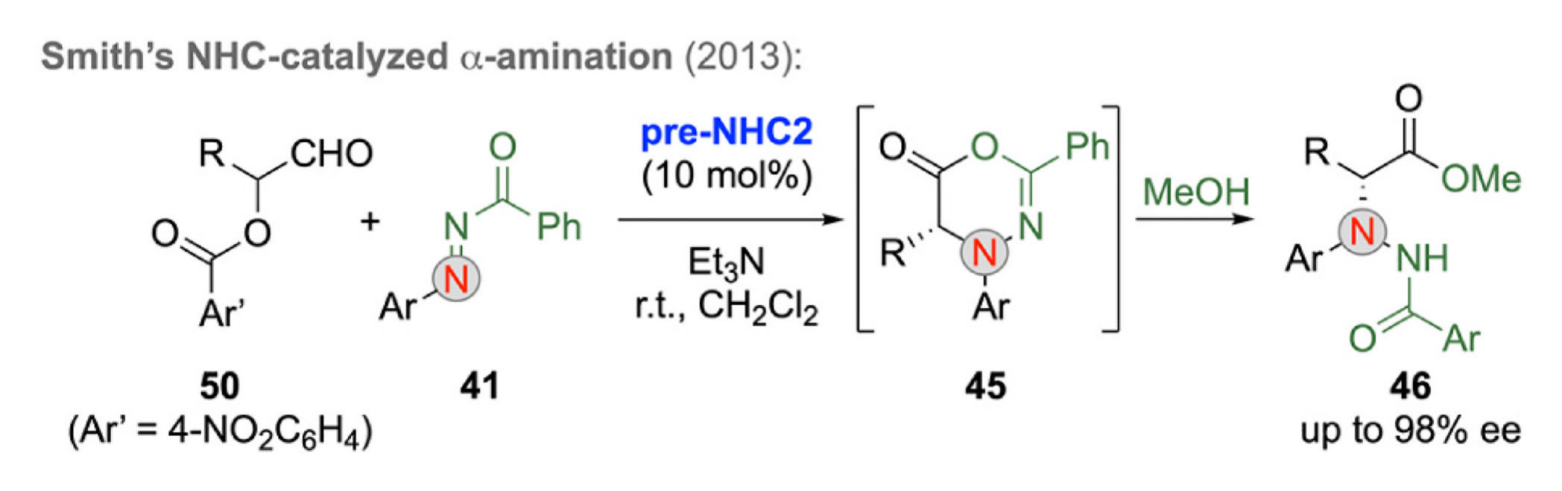
NHC-catalyzed α-amination of α-aroyloxyaldehydes.

As mentioned in the introduction, C1-LB enolates can also be accessed by starting from carbaldehydes containing a leaving group in the α-position (compare with Scheme 1). In 2013, Smith’s group reported the use of α-aroyloxyaldehydes **50** as C1-LB enolate precursors under NHC catalysis, which allowed for asymmetric [4 + 2]-cycloadditions with diazo-compounds **41** (Scheme 14) and subsequent transformations in analogy to the above described ITU process (Scheme 13) [37].

## 5 Conclusion

Asymmetric C1-Lewis base enolate chemistry has been established as a powerful covalent organocatalysis strategy to access a diverse array of valuable carboxylic acid derivatives in a stereoselective manner. As outlined in this minireview, over the course of the last two decades this concept has been very successfully utilized for various α-heterofunctionalization reactions. Different catalyst classes, i.e. chiral Cinchona alkaloids, N-heterocyclic carbenes, pyridine derivatives, and isothioureas have been used for the activation and control of starting materials like carboxylic acid halides, free carboxylic acids, ketenes, activated esters, or suited carbaldehydes, thus resulting in a multitude of divers and broadly applicable methods. Interestingly, while asymmetric α-halogenations have been investigated very successfully allowing for the introduction of a variety of conceptually different approaches so far, α-chalcogenations and α-pnictogenations have so far less generally been established and are limited to α-oxygenations and α-aminations so far. On the other hand, analogous α-sulfanylations/selenations are yet missing. One aspect that turned out to be crucial herein is the identification of suited electrophilic heteroatom-transfer reagents that show sufficient reactivity and which also allow for high enantioselectivities under these covalent catalysis conditions, as exemplified for our failed attempts to establish enantioselective α-sulfanylations and selenations (Scheme 11). Thus, it has to be emphasized that despite of the recent progress that has been made for α-halogenations, α-oxygenations and α-aminations, this field is still an emerging one, which will require future studies to overcome the existing limitations by introducing new catalyst motives and/or establishing alternative electrophilic heteroatom transfer reagents.

## Data Availability

This is a review article. Everything covered herein was cited in the reference section.
